# Antiviral response mechanisms in a Jamaican Fruit Bat intestinal organoid model of SARS-CoV-2 infection

**DOI:** 10.21203/rs.3.rs-2340919/v1

**Published:** 2022-12-12

**Authors:** Marziah Hashimi, Thomas Sebrell, Jodi Hedges, Deann Snyder, Katrina Lyon, Stephanie Byrum, Samuel G Mackintosh, Michelle Cherne, David Skwarchuk, Dan Crowley, Amanda Robison, Barkan Sidar, Anja Kunze, Emma Loveday, Matthew Taylor, Connie Chang, James Wilking, Seth Walk, Tony Schountz, Mark Jutila, Diane Bimczok

**Affiliations:** Montana State University; Montana State University; Montana State University; Montana State University; Montana State University; Univ. of Arkansas for Medical Sciences; University of Arkansas for Medical Sciences; Montana State University; Montana State University; Montana State University; Montana State University; Montana State University; Montana State University; Montana State University; Montana State University; Montana State University; Montana State University; Montana State University; Colorado State University; Montana State University; Montana State University

## Abstract

Bats are natural reservoirs for several zoonotic viruses, potentially due to an enhanced capacity to control viral infection. However, the mechanisms of antiviral responses in bats are poorly defined. Here we established a Jamaican fruit bat (JFB) intestinal organoid model of severe acute respiratory syndrome coronavirus-2 (SARS-CoV-2) infection. JFB organoids were susceptible to SARS-CoV-2 infection, with increased viral RNA and subgenomic RNA detected in cell lysates and supernatants. Gene expression of type I interferons and inflammatory cytokines was induced in response to SARS-CoV-2 but not in response to TLR agonists. Interestingly, SARS-CoV-2 did not lead to cytopathic effects in JFB organoids but caused enhanced organoid growth. Proteomic analyses revealed an increase in inflammatory signaling, cell turnover, cell repair, and SARS-CoV-2 infection pathways. Collectively, our findings suggest that primary JFB intestinal epithelial cells can mount a successful antiviral interferon response and that SARS-CoV-2 infection in JFB cells induces protective regenerative pathways.

## Introduction

Bats are considered important natural reservoirs for a variety of emerging zoonotic viruses that cause several illnesses in humans and other mammals ^1^, including severe acute respiratory syndrome coronavirus (SARS-CoV), Middle East respiratory syndrome coronavirus (MERS-CoV), Hendra virus, Ebola virus, and Marburg virus ^2, 3, 4, 5, 6^. The ongoing COVID-19 pandemic is caused by severe acute respiratory coronavirus-2 (SARS-CoV-2) ^7^ which also is thought to have originated in bats. This hypothesis is based on multiple studies that demonstrated a high level of genetic similarity between SARS-CoV-2 and several bat-borne coronaviruses such as RatG13 (96.1% identity ^7^) and BANAL-52 (96.8% identity ^8^), which have been detected in bat feces. Studies from a number of different bat species have shown that bat viruses, including coronaviruses, achieve long-term colonization of intestinal tissues ^9^. In a study by Watanabe *et al.* on wild bats captured in the Philippines ^10^, enteric coronaviruses were detected in > 50% of the animals, but clinical signs of disease were absent. Similarly, Subudhi *et al.* found that 30% of North American little brown bats harbored coronaviruses in their intestines but did not display any signs of illness ^11^. Tong *et al.* analyzed rectal swabs and intestinal tissues from asymptomatic fruit bats in Peru and identified a novel influenza A virus, H18N11 ^12^. In contrast to bats, where gastrointestinal infections with eukaryotic viruses are frequent and are commonly asymptomatic ^13^, a similar colonization of the human gut with non-pathogenic eukaryotic viruses has not been reported, pointing to species-specific mechanisms ^14^.

Studying coronavirus infection in the GI tracts of bats is difficult, since few institutions maintain bat colonies for *in vivo* infection experiments, and cell lines from the GI tract of bats are not available, limiting *in vitro* analyses ^15, 16^. Organoid cultures have untapped potential as a model to study the mechanisms of viral infection in bat cells *in vitro* ^17, 18^. Organoids are permanent three-dimensional cultures that replicate the physiological and functional characteristics of their tissues of origin and that allow controlled studies of complex primary GI epithelial tissues *in vitro* ^19^. Organoids from various human and murine tissues have been developed from tissue-derived stem cells and have been successfully used to investigate a wide range of disease processes, including viral infections ^17, 18, 20, 21^. Importantly, growth conditions for GI organoids appear similar across multiple species ^22^. Two previous studies have described the generation of intestinal organoid cultures from bat species ^23, 24^. Intestinal organoids developed from Chinese horseshoe bats, *Rhinolophus sinicus*, showed susceptibility to SARS-CoV-2, but lacked long-term active proliferation past 4–5 weeks ^25^. Intestinal organoids derived from Leschenault’s rousette, *Rousettus leschenaultii.* showed susceptibility to *Pteropine* orthoreovirus, but not SARS-CoV-2 ^23^. However, neither of these studies evaluated the cellular antiviral mechanisms of bat organoid tissues ^23, 25^.

The hypothesis that altered IFN responses in bats compared to other species promote increased viral tolerance is a central paradigm in bat immunology ^26, 27^. In Australian black flying foxes (*P. alecto),* a high level of constitutive IFN-α expression was detected, which has led to the concept that an “always on” IFN signaling system in bats can effectively suppress viral replication and prevent disease early on after infection ^26, 28^. Increased basal gene expression in bats also was described for several other genes involved in innate viral recognition and response, including IRF1, IRF3 and IRF7 ^29^ and the ISG oligoadenylate synthase 1 (OAS1) ^30^. Conversely, dampened activation of stimulator of IFN genes (STING), a nucleic acid sensor involved in the regulation of IFN expression upon viral infection, also has been reported ^31, 32^. Importantly, these characteristics of the IFN system appear to be unique to particular bat species, pointing to a need for more detailed analyses.

Jamaican fruit bats (JFBs) are thought to be natural carriers of zoonotic viruses such as rabies, West Nile and dengue viruses and are one of the most common bats in the Americas, making them a relevant species for experimental investigations ^6, 33, 34, 35, 36, 37^. JFBs also are susceptible to experimental infection with Zika virus and MERS-CoV ^6, 36^. Based on the recently annotated genome ^38, 39^, JFBs have one interferon (IFN)-β gene, five IFN-α genes, and five IFN-ω genes. Multiple interferon regulatory factors (IRFs) have also been identified, making JFBs a tractable model system for studies of antiviral immunity.

Here we established and characterized gut organoids from JFBs to study the susceptibility and immune response of the JFB intestinal epithelium to SARS-CoV-2 infection. We found that JFB intestinal epithelial cells supported modest viral replication that did not result in the release of infectious virions or cytopathic effects. Contrary to the “always on” paradigm for antiviral interferon responses in bats ^28^, the organoids mounted a robust interferon response to infection with active SARS-CoV-2. Proteomics and pathway analysis revealed that the JFB organoid proteome profiles matched profiles found in other SARS-CoV-2 infection studies and that SARS-CoV-2 infection activated innate inflammatory and cellular repair responses in this model system.

## Results

### Development and Characterization of JFB Gastrointestinal Organoids.

**Figure 1:**
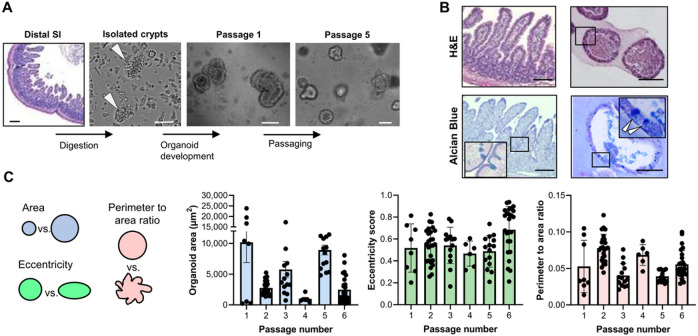
Development and culture of gastrointestinal organoids from Jamaican fruit bats. (**A**) Organoid derivation from Jamaican fruit bat (JFB) distal small intestine. Tissue of origin, isolated intestinal crypts and formed organoids are shown. Scale bar: 200 μm for distal SI, others are 50 μm. (**B**) Morphology of distal SI tissue (left) and distal small intestinal organoids (right). Formalin-fixed, paraffin-embedded sections were stained with H&E (top row) or Alcian Blue (bottom row). High magnification insets show columnar cell shape and morphology of mucus-secreting goblet cells. Bars: 100 μm. (**C**) Size and morphology of distal SI organoids were analyzed over six consecutive passages using OrganoSeg ^41^. Dots: individual organoids (n≥6); bars: mean ± SD.

We established JFB gastrointestinal organoid cultures from fresh and cryopreserved stomach and from proximal and distal small intestine (Fig. 1A and **Supplemental Fig. 1A, B**). Organoids formed within one day of crypt/gland isolation and were successfully maintained in a simple growth medium containing DMEM and 50% L-WRN-conditioned medium (**Supplemental Fig. 1C**).The murine noggin, R-spondin, and Wnt3a secreted by the L-WRN cells ^40^ show protein sequence similarities of 98%, 86%, and 99% with the orthologous JFB proteins, suggesting that these factors would be active in JFB cells (**Supplemental Fig. 2**).

Established JFB organoids mimicked the epithelial structure of JFB gastrointestinal tissue, with a simple columnar epithelium, a basal nucleus and a defined luminal space (Fig. 1B, **Supplemental Fig. 3A, B**). Mucus-secreting cells were present in organoids derived from distal small intestine and stomach, but were rare in proximal small intestinal organoids, consistent with the cellular composition of the respective tissues of origins (Fig. 1B, **Supplemental Fig. 3A, B**). Morphometric analysis with OrganoSeg ^41^ showed that organoid size varied between different passages, but with no clear trends, and organoid shape also did not change significantly over six consecutive passages (Fig. 1C). JFB organoids were maintained for at least 30 passages (> 6 months), and also were successfully cryopreserved and re-cultured from cryopreserved stocks (data not shown).

**Figure 2:**
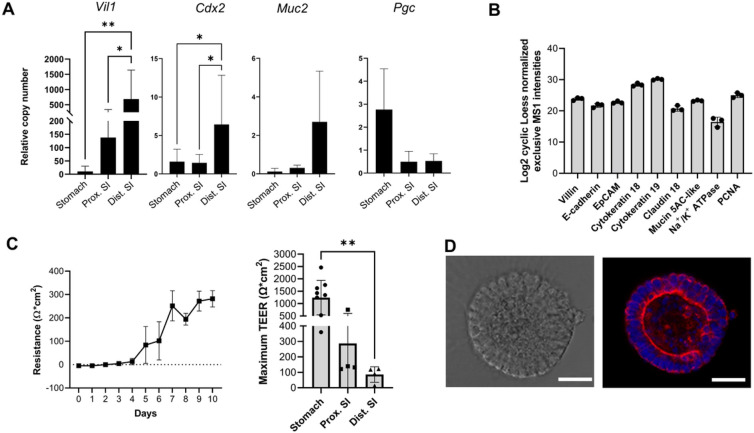
Characterization of gastrointestinal organoids from Jamaican fruit bats. (**A**) Conservation of tissue-specific gene expression patterns in JFB organoids derived from stomach, proximal and distal SI. Pooled qRT-PCR data from n=4 established organoid lines (p2-10) are shown; mean ± SEM. Statistical analyses were performed using ANOVA with Tukey’s multiple comparisons test; *P≤0.05, **P≤0.01. (**B**) Expression of select intestinal epithelial cell-specific proteins. JFB distal SI organoids (3 technical replicates) were lysed and processed for data-independent acquisition mass spectrometry. Individual datapoints and mean ± SD. (**C**) Barrier function of JFB organoid cells cultured on transwell inserts for 10 days. Representative data of distal small intestinal (SI) organoids with mean ± SEM of triplicate wells (left) and pooled data from four experiments (right) with gastric, proximal SI and distal SI organoids are shown. (**D**) Confocal imaging reveals polarized expression of cytokeratin in JFB organoids. Left: brightfield image, right: single Z-plane; pan-cytokeratin-red, nuclei-blue. One representative of 3 experiments. Bars: 25 μm.

We next performed transcriptional analysis of the organoids to confirm tissue-specific gene expression patterns. The distal and proximal intestinal organoids expressed the intestine-specific genes *Vil1, Cdx2*, and *Muc2*, while the gastric organoids showed increased expression of the chief cell marker pepsinogen C (*Pgc*) with low expression of *Vil1, Cdx2* and *Muc2* (Fig. 2A). Since no specific reagents for JFB cells are available, we next performed an unbiased proteome analysis using data-independent acquisition (DIA) mass spectrometry using organoids from JFB distal small intestine. Several key proteins characteristic of small intestinal epithelial cells in other mammals such as villin, E-cadherin, keratin 18 and 19, Na^+^/K^+^ ATPase, claudin 18, and a mucin (MUC5AC-like) were detected (Fig. 2B) ^42^, confirming the identity of the intestinal organoids. Measurement of transepithelial electrical resistance (TEER) across organoid monolayers seeded on transwell inserts showed that the gastrointestinal organoids established a physiological epithelial barrier, with the stomach having the highest TEER compared to the intestinal organoids (Fig. 2C). Confocal imaging analysis of cytokeratin expression confirmed epithelial cell polarization and correct inside-in orientation of the organoids (Fig. 2D). Collectively, these analyses demonstrate that gastrointestinal organoids from JFBs replicate key features of the gastrointestinal epithelium.

### Infection of JFB distal organoids with SARS-CoV-2 leads to replication of viral genomes.

**Figure 3:**
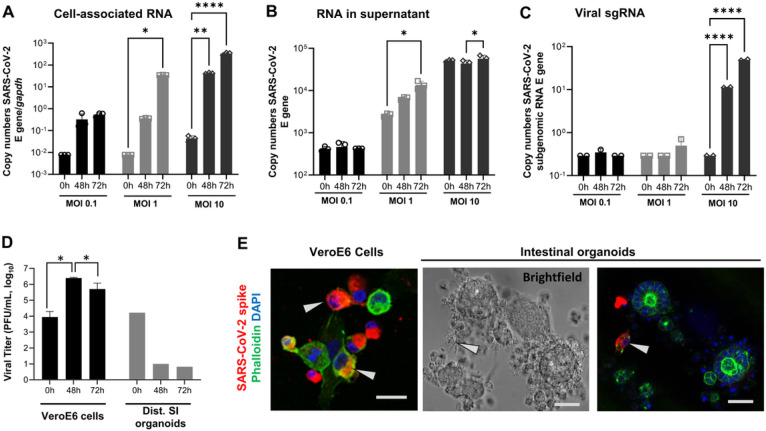
Replication of SARS-CoV-2 in JFB intestinal organoids. Dissociated JFB distal intestinal organoids were inoculated with SARS-CoV-2 (strain USA-WA1/2020) for 2 h or were mock-treated and then were washed and re-embedded in Matrigel. At 48 and 72 h post infection, RNA was extracted from (**A**) the organoids and (**B**) the culture supernatants, and replication of SARS-CoV-2 was analyzed by quantitative real-time PCR (qRT-PCR) for the envelope (E) gene using the standard curve method. (**C**) RNA extracted from the organoids was analyzed for viral sgRNA (E gene) using a leader-specific primer. (**D**) Supernatants from SARS-CoV-2 infected organoids or Vero E6 cells were analyzed by plaque assay for the presence of infectious SARS-CoV-2. (A-D) Panels show data from one representative out of three to four independent experiments with two or three technical replicates as mean ± SEM, analyzed by ANOVA with Dunnett’s or Tukey’s multiple comparisons test; *P≤0.05, **P≤0.01, ***P≤0.0001. (**E**) SARS-CoV-2 protein detection in isolated epithelial cells, but not in intact JFB intestinal organoids. Organoids or Vero E6 cells were fixed and permeabilized at 48 h post SARS-CoV-2 infection (MOI 10) and then were stained with DAPI (blue), phalloidin (green) and a monoclonal antibody to SARS-CoV-2 spike protein (red). Arrows point out cells containing SARS-CoV-2 spike protein. Data are representative of three independent experiments. Scale bar= 25 μm.

**Figure 4:**
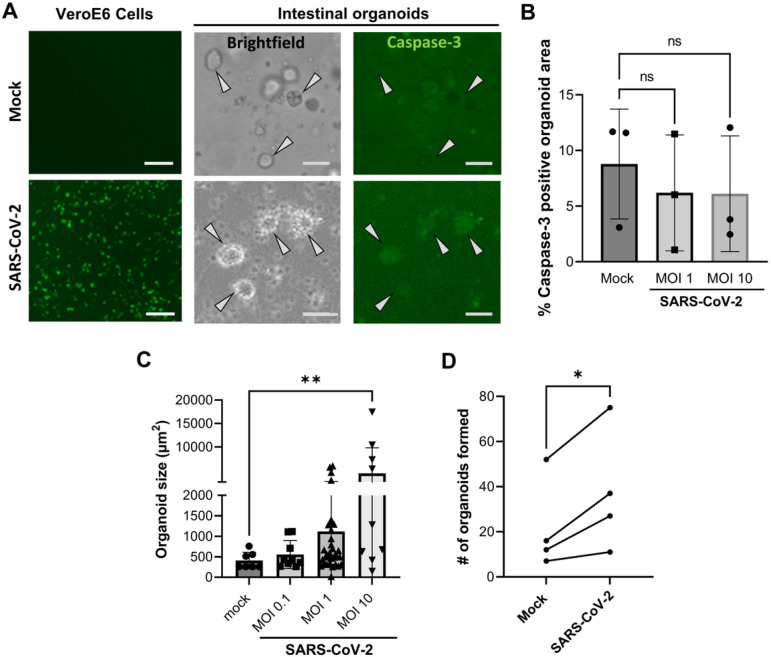
Increased growth of JFB organoids infected with SARS-CoV-2. Dissociated JFB distal intestinal organoids or Vero E6 cells were mock-inoculated or were infected with SARS-CoV-2 at an MOI of 1 or 10, as described above, with NucView^®^ 488, a cell membrane-permeable fluorogenic caspase-3 reporter, added to the medium. (**A**) At 48 h post infection, the cells were imaged using fluorescence and phase contrast (brightfield) microscopy. Scale bars = 50 μm. (**B**) ImageJ was used to quantitate NucView^®^ fluorescence based on pixel counts in thresholded digital images of manually selected organoids. Individual data points, mean ± SD of one representative of four independent experiments with three technical replicates is shown, data were analyzed by Student’s *t* test. (**C**) Organoid size in SARS-CoV-2-infected organoid cultures after 48 h was determined on brightfield images using ImageJ. Individual data points, mean ± SD of one representative of five independent experiments with three technical replicates is shown, data were analyzed by ANOVA with Tukey’s multiple comparisons test; **P≤0.01. (**D**) Number of detected organoids in random brightfield images from mock-infected and SARS-CoV-2 infected JFB organoid cultures (MOI 10, 48 h). Pooled data from four independent experiments; Student’s *t* test, *P≤0.05.

To determine whether the JFB intestine supports SARS-CoV-2 infection, organoids were dissociated and then inoculated with SARS-CoV-2 at MOIs of 0.1, 1 and 10. We selected distal intestinal organoids for these experiments, based on several previous publications that demonstrated SARS-CoV-2 replication in human ileal organoids ^43, 44, 45^. Quantitative PCR analysis of viral genomes in JFB organoid cell lysates revealed a significant, concentration-dependent increase (> 1 log, *P* < 0.05) in SARS-CoV-2 gene E RNA at 48 and 72 hours post infection (hpi, Fig. 3A). The SARS-CoV-2 PCR in culture supernatants showed a similar increase at an MOI of 1 at 48 hpi (Fig. 3B). Importantly, significant expression of subgenomic (sg)RNA (> 2 log-fold above baseline) for gene E indicating active viral replication in the organoids also was identified ^46^, albeit at low levels (Fig. 3C). However, plaque assays performed on the culture supernatants failed to detect the presence of infectious SARS-CoV-2 above baseline values derived from the inoculum, suggesting incomplete or ineffective viral replication or failure to secrete progeny virus (Fig. 3D). Culture of the organoids in differentiation medium with reduced Wnt3a or as 2D monolayers did not alter these results (data not shown). Notably, SARS-CoV-2 incubation in medium for 48 h did not impact detection of viral copy numbers by PCR, but did reduce the viral titer measured by plaque assay by > 1 log-fold, suggesting a loss of infectivity over time (**Supplemental Fig. 4**). Interestingly, immunofluorescence analysis of SARS-CoV-2 spike protein in infected JFB organoids revealed only a few positive cells, and these cells were not associated with morphologically intact organoids (Fig. 4E).

## Lack of cytopathic effect but increased growth in SARS-CoV-2 infected JFB organoids

We also evaluated the cell viability of JFB distal intestinal organoids following SARS-CoV-2 infection by measuring caspase-3 activity with NucView^®^^47^ (Fig. 4A). In Vero E6 cells, infection with SARS-CoV-2 induced a strong upregulation of caspase-3, consistent with the well-characterized cytopathic effect of the virus in this cell type. A small number of apoptotic cells were present in all JFB organoid cultures, likely due to physiological cell turnover. However, in contrast to observations in *Rhinolophus sinicus* organoids ^25^, SARS-CoV-2 did not appear to have a cytopathic effect in JFB organoids (Fig. 4A,B), since the proportion of apoptotic cells did not change upon infection. Interestingly, SARS-CoV-2 caused a significant increase in organoid size and in the number of organoids that had re-formed from single cells after 48 h of infection (Fig. 4C,D), indicating that viral infection triggered increased cell proliferation in the bat intestinal epithelium.

### SARS-CoV-2 induces expression of type I interferons and proinflammatory cytokines in JFB organoids.

**Figure 5:**
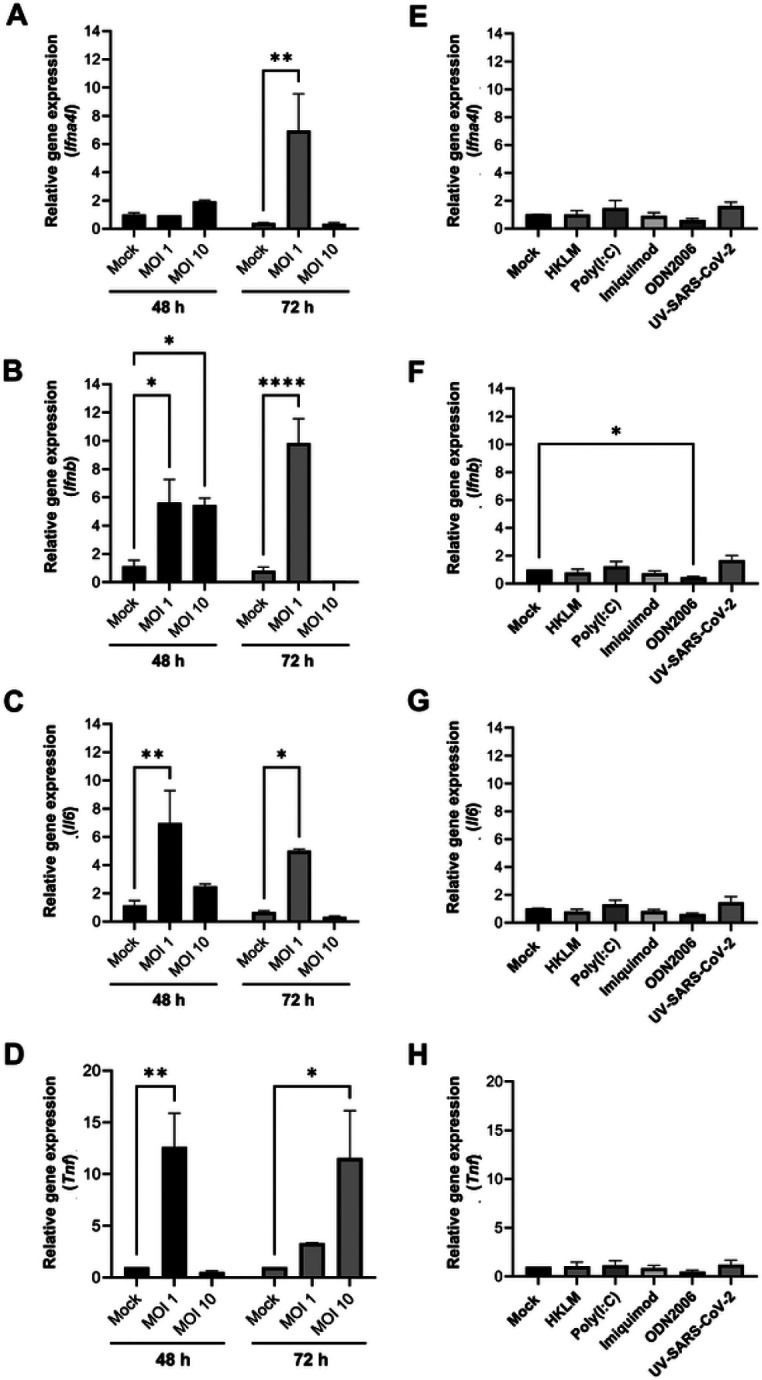
JFB distal intestinal organoids express antiviral and pro-inflammatory genes in response to infection with SARS-CoV-2. **(A-D)** Dissociated JFB distal intestinal organoids were infected with active SARS-CoV-2 at an MOI of 1 or 10. The unbound virus was washed off, and the cells were re-plated in Matrigel. After 48 or 72 h, the RNA was extracted from the cells to evaluate genes expression via quantitative real-time PCR (qRT-PCR). Data from one representative out of four independent experiments run in triplicate are shown as mean ± SEM. (**E-H):** Organoids were treated with UV-inactivated SARS-CoV-2 at 10 μg/mL, or with a panel of TLR agonists (TLR2: heat-killed *L. monocytogenes*, HKLM; TLR3: low MW poly I:C; TLR7: imiquimod, TLR9: ODN2006) and then were analyzed by qRT-PCR 48 h after stimulation. Pooled data for three independent experiments; mean ± SEM are shown. Graphs show gene expression of (**A, E**) *Ifna4l* (IFNα 4-like), (**B, F**) *Ifnb* (IFN-β), (**C, G**) *Il6* (IL-6) and (**D, H**) *tnf* (TNF-α). All data were analyzed using the 2^(−ΔΔCt)^ method with *gapdh* as a housekeeping gene and are expressed as fold change relative to the mock-infected control. ANOVA with Dunnett’s multiple comparisons test; *P≤0.05, **P≤0.01, ***P≤0.0001.

Unique characteristics of the interferon (IFN) system have been linked to the increased viral tolerance observed in many bat species ^26^. Therefore, we used quantitative RT-PCR to analyze gene expression of type I interferons and proinflammatory cytokines in JFB distal small intestinal organoids following 48 h exposure to SARS-CoV-2. As shown in Fig. 5A, expression of type I interferon *Ifna4l* was upregulated at 48 hpi with an MOI of 10, while an MOI of 1 caused significant upregulation of the gene at 72 hpi. Gene expression of *Ifnb* also was significantly increased with both MOIs at 48 hpi and remained elevated with the lower viral dose at 72 hpi (Fig. 5B). Type III IFNs are known to play a role in mucosal antiviral immunity and SARS-CoV-2 infection and also may have unique functions in bats ^48, 49, 50^. However, the type III IFN loci in JFBs are poorly annotated ^38, 39^, and we unable to generate functional primers based on the published genome. Interestingly, organoid infection with SARS-CoV-2 at an MOI of 1 significantly increased expression of the proinflammatory cytokines *Tnf* and *Il6* at 48 hpi, and *Il6* remained elevated at 72 hpi (Fig. 5C,D). The above data suggest that JFB distal organoids exhibited an anti-viral and proinflammatory response to SARS-CoV-2 infection.

To determine whether active viral infection was responsible for the observed induction of antiviral and inflammatory genes, or whether gene expression was induced by unspecific activation of pattern recognition receptors, we also treated the JFB organoids with a panel of TLR agonists targeting TLR2, 3, 7, and 9 and with UV-inactivated SARS-CoV-2 for 48 h. Notably, stimulation with TLR2/1 and TLR3 agonists led to increased expression of interferon and inflammatory cytokines 6 h post inoculation (**Supplemental Fig. 5**). However, no significant upregulation of these genes was observed with any of the stimuli at 48 h (Fig. 5E-H). These observations suggest that active infection with SARS-CoV-2 is required for sustained upregulation of antiviral and inflammatory gene expression.

## Impact of SARS-CoV-2 infection on the JFB intestinal epithelial cell proteome

**Figure 6:**
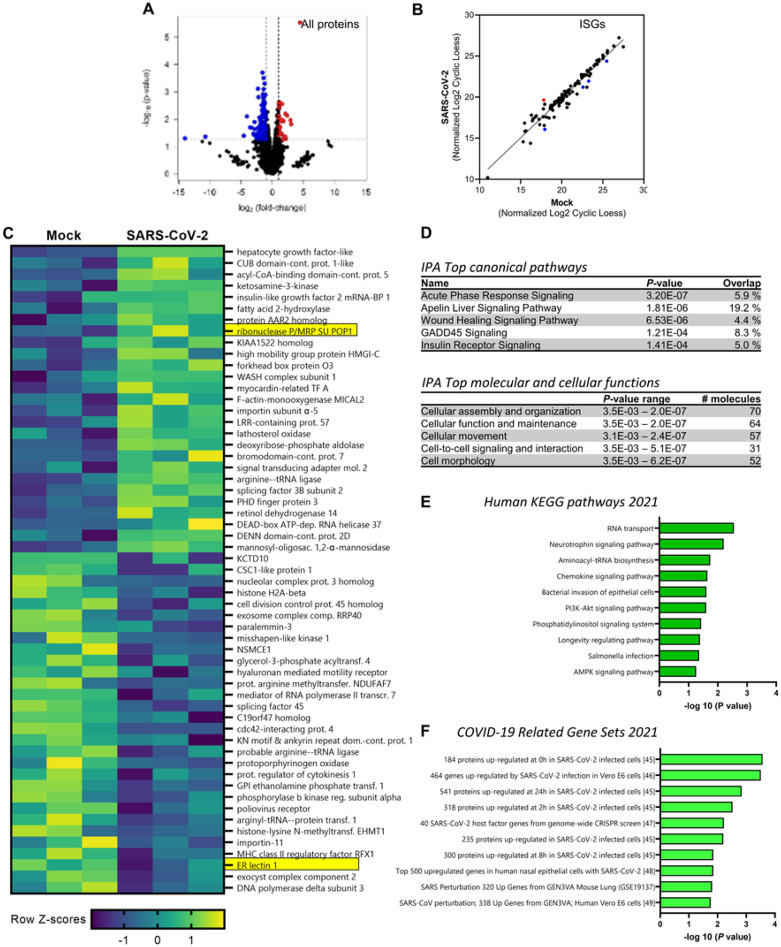
Proteome analysis of SARS-CoV-2-infected JFB organoids at 48 h. JFB distal SI organoids were infected with SARS-CoV-2 at an MOI of 10 or underwent mock treatment for 48 h and then were lysed and processed for data-independent acquisition (DIA) mass spectrometry. N=3 replicates from one organoid line were analyzed. (**A**) Volcano plot showing all detected proteins and protein isoforms. Proteins with significantly increased or decreased expression (≥2-fold change; *P*≤0.05) are shown in red and blue. (**B**) Expression of interferon-stimulated genes (ISGs), identified based on OhAinle et al. (2018) ^53^, in mock-infected and SARS-CoV-2 infected JFB organoids. Proteins with significantly increased or decreased expression (≥2-fold change; *P*≤0.05) are shown in red and blue. (**C**) Heatmap showing relative change (Z-scores) of all 27 significantly upregulated proteins and of the top 30 downregulated proteins (*P*≤0.05). Data from triplicate cultures are shown. Protein function was determined using UniProtKB (*H. sapiens)*. Significantly regulated ISGs are highlighted in yellow. (**D**) IPA analysis showing top regulated canonical signaling pathways (top) and molecular and cellular functions (bottom) activated in SARS-CoV-2 infected JFB organoids. (**E,F**) Enrichr pathway analysis using (**E**) the 2021 human KEGG pathway database and (**F**) the 2021 COVID-19 related gene sets. Pathways were ranked based on combined score ranking.

A quantitative proteomic workflow based on data-independent acquisition (DIA) mass spectrometry was used to perform a comprehensive analysis of the cellular responses of JFB organoids to SARS-CoV-2 infection. The DIA analysis of SARS-CoV-2-infected and mock infected enteroids after 48 h yielded a total of 8,321 proteins and protein isoforms, based on protein FASTA files retrieved from the *A. jamaicensis* reference genome ^51, 52^. Interestingly, all detected proteins were present in both experimental conditions. A comparative analysis of mock-infected and SARS-CoV-2 infected JFB organoids revealed 63 upregulated and 155 downregulated proteins, including isoforms, with a ≥ 2-fold change at *P* ≥ 0.05 (Fig. 6A **and Supplemental Table 1**). To better understand antiviral responses in the JFB intestine, we next compared the identified proteins to a comprehensive list of human interferon-stimulated genes (ISGs, **Supplemental Table 2**) ^53^. Interestingly, 100 of all identified JFB proteins could tentatively be classified as ISGs. However, only one of the ISG proteins, ribonucleases P/MRP protein subunit POP1 (POP1), was significantly upregulated in response to SARS-CoV-2, while four ISG proteins (ERLEC1, CFB, ARMCX3 and ITIH2) were significantly downregulated (Fig. 6B). Overall, top upregulated proteins, based on fold change in abundance, were hepatocyte growth factor-like protein/macrophage stimulatory protein (HGFL/MST1), CUB domain-containing protein 1-like, acyl-CoA-binding domain-containing protein 5 (ACBD5), ketosamine-3-kinase (KT3K) and insulin-like growth factor 2 mRNA-binding protein 1 (IGF2BP1, Fig. 6C). Top down-regulated proteins included BTB/POZ domain-containing adapter for CUL3-mediated RhoA degradation protein 3 (KCTD10), CSC1-like protein 1 (TMEM63A), nuclear complex protein 3 homologue, histone H2A-β, and cell division complex protein 45 homologue (CDC45) (Fig. 4D). Several of these proteins are involved in regulation of cell turnover and posttranslational modifications. We next performed Ingenuity Pathway Analysis (IPA) and Enrichr analysis ^54^ to assess more complex functional changes induced by SARS-CoV-2. IPA revealed acute phase response signaling, a key innate pathway triggered by infection and injury, as the most significantly regulated pathway, followed by Apelin liver signaling ^55^, which is involved in intestinal inflammation, repair, and wound healing (Fig. 6D). Top regulated cellular functions were cell assembly, organization, maintenance, movement, signaling and morphology (Fig. 6D). These findings suggest that SARS-CoV-2 triggers regenerative response pathways, consistent with the increased organoid size observed in the SARS-CoV-2-infected compared to mock-infected cultures. Similarly, Enrichr identified significant upregulation of pathways associated with cell viability and differentiation, such as PI3/AKT signaling and the longevity regulating pathway, along with signatures associated with intestinal epithelial infection and chemokine signaling when using the human 2021 KEGG pathways database (Fig. 6E). Importantly, Enrichr analysis also found multiple significant matches for protein signatures that were previously found to be upregulated in SARS-CoV-2 infection in various experimental systems ^56, 57, 58, 59, 60^ (Fig. 6F). Overall, the proteomics analysis points to the activation of innate inflammatory and regenerative pathways along with characteristic COVID-19 signatures upon SARS-CoV-2 infection of the JFB intestinal epithelium.

## Discussion

In this study, we established and characterized organoid cultures from the proximal and distal small intestine and stomach of JFBs. Using this model, we investigated the response of JFB intestinal epithelial cells to infection with SARS-CoV-2. Considering that JFBs are susceptible to MERS-CoV, Zika virus, and rabies virus ^6, 36, 37^, we evaluated the susceptibility of the JFB distal intestinal organoids to SARS-CoV-2. Notably, JFBs are not thought to be natural carriers of SARS-CoV-2, and no studies on *in vivo* infection of JFBs with SARS-CoV-2 have been published to date. Considering the vast number and associated genetic diversity of bat species it is not surprising that SARS-CoV-2 infection experiments in other bat species have yielded conflicting results. In Egyptian fruit bats (*Rousettus aegyptiacus),* transient asymptomatic respiratory tract infection with viral replication in lung and trachea and oral and fecal shedding was achieved upon experimental SARS-CoV-2 inoculation ^61^. Conversely, American big brown bats (*Eptesicus fuscus*) appeared resistant to infection with SARS-CoV-2 ^62^. Likewise, intestinal organoids derived from two different bat species responded differently to SARS-CoV-2 infection. Organoids from Chinese horseshoe bats, where SARS-CoV-2-like virus has been detected ^7^, produced infectious SARS-CoV-2 virions at similar levels as human intestinal organoids ^25^. In contrast, intestinal organoids from Leschenault’s rousette bats (*Rousettus leschenaultii*) failed to support SARS-CoV-2 replication ^23^. Interestingly, PCR analysis revealed a significant increase in viral and sgRNA in the JFB distal organoids at 48 and 72 hpi, which demonstrates initiation of viral replication in the organoids. SARS-CoV-2 genomes also were significantly increased in organoid culture supernatants. Using immunohistochemistry, we detected SARS-CoV-2 spike protein in individual cells, but not in morphologically intact JFB organoids. This observation may reflect shedding of viable virus-infected cells from the epithelial monolayer, as described for other viral infections ^63^. We also did not detect infectious virions in organoid cells or supernatants using plaque assays in VeroE6 cells, suggesting that JFB intestinal organoids support incomplete SARS-CoV-2 infection. A similar limited and incomplete replication of SARS-CoV-2 was also reported in cell lines from several different bat species, even after transduction with human ACE2, in a recent study by Aicher *et al.* ^64^. However, the presence of sgRNA and of SARS-CoV-2 protein in some cells suggest that entry and replication of the virus did occur in the JFB organoids. This interpretation is consistent with a study by Yan *et al.* that predicted a moderate ability of SARS-CoV-2 to infect JFB cells based on the protein sequence of the SARS-CoV-2 receptor ACE2 ^65^ and our unpublished observations of ACE-2 gene expression on the JFB organoids. Loss of the furin cleavage site in the WA01 reference stain of SARS-CoV-2 also may have had an impact on the efficacy of infection ^66^. Further experiments are needed to evaluate at which stage of viral replication cycle SARS-CoV-2 replication stalls in the JFB organoid model and whether JFBs are permissive to SARS-CoV-2 infection *in vivo*. Notably, many previous studies on viral infection in bats have relied solely on viral nucleic acids to measure infection ^5, 10, 11, 12, 35, 62^. Therefore, it is difficult to assess whether the failure to detect replication-competent virions was unique to our infection model.

Our results demonstrate that active SARS-CoV-2 virus induced a robust anti-viral immune response, with increased expression of IFN-α and IFN-β at 48 h after SARS-CoV-2 infection. This strong induction of interferons in response to viral infection was surprising, since the current paradigm is that the interferon system in bats is constitutively active, based on studies in Australian black flying foxes (*P. alecto)*^26, 67^. Conversely, potent interferon responses were detected in serotine bats (*Eptesicus serotinus*) and David’s myotis bat cells upon SARS-CoV-2 infection ^64^. These observed differences point to species-specific immune system characteristics in bats, consistent with the high level of genetic diversity in the order Chiroptera, which comprises over 1,400 species.

The lack of a cytopathic effect in SARS-CoV-2-infected JFB organoids was an intriguing observation. It has been shown that SARS-CoV-2 causes neither apoptotic nor necrotic cell death in the gastrointestinal tract of infected human patients. Whether SARS-CoV-2 impacts viability of organoid cultures is still a matter of debate. Lamers *et al.* ^43^ observed increased apoptotic cell death in human enteroids at 60 hpi, and Zhou *et al.* ^25^ state that both human and horseshoe bat enteroids developed a cytopathic effect after SARS-CoV-2 inoculation. Conversely, studies by Stanifer *et al.* ^48^ and Zang *et al.* ^68^ did not describe increased cell death in human SARS-CoV-2 infected enteroids. Data from several studies in bat cells suggest that heightened IFN responses in these cultures may prolong viral infection by limiting pathogen-induced cell death through induction of anti-apoptotic genes including BCL-2 and PMAIP1 ^35, 69^. While we did not detect an upregulation in anti-apoptotic factors in our proteome screen of SARS-CoV-2-infected JFB organoids, we found a significant upregulation of pathways associated with cell growth and repair, including apelin liver signaling and wound healing signaling. These observations were consistent with the increase in organoid size and organoid formation that we detected by microscopic analysis and indicate activation of growth and repair pathways in response to SARS-CoV-2 infection. Taken together, our findings suggest that bat organoids activate protective repair pathways upon viral infection that may enable the bats to tolerate viral infection in the absence of tissue damage and associated clinical signs.

We used a proteomics approach to gain deeper insights into the cellular responses induced in the SARS-CoV-2 infected JFB intestinal epithelium. Notably, our study was the first, to our knowledge, to use a DIA-based proteomics approach with JFB cells. Our analysis confirmed the identity of the organoids as small intestinal epithelial cells based on expression of key enterocyte markers. Consistent with the increased gene expression of pro-inflammatory cytokines in SARS-CoV-2-infected JFB organoids that we detected, inflammatory pathways including the acute phase response and chemokine signaling also were induced at the protein level. Conversely, although SARS-CoV-2 infection induced expression of type I interferon transcripts in JFB organoids, no significant increase in ISGs was detected on the protein level. This lack of ISG regulation is inconsistent with proteomics results obtained in SARS-CoV-2-infected human Calu-3 cells, which showed a strong induction of the antiviral ISG signature ^70^, and may reflect a JFB-specific disconnect between transcriptional activation of interferons and downstream ISGs that warrants further studies. Alternatively, downregulation of ISG proteins may have been caused by active downregulation of antiviral ISG pathways by SARS-CoV-2 accessory proteins. Importantly, Enrichr analysis also revealed that some of the activated pathways matched those identified by other studies on SARS-CoV-2 infection. Notably, there are several limitations to the proteomics approach undertaken in our study. First, the non-targeted DIA approach may not be sensitive enough to identify strongly regulated targets with a low overall expression level ^71^. Second, an annotated proteome of the JFB is currently not available and thus had to be inferred from the genome, which may lead to misidentified proteins. Lastly, pathway analysis was based on human databases, which again may miss JFB-specific signaling pathways.

Importantly, we successfully validated JFB organoids as an experimental tool and demonstrated that these JFB organoids can be maintained long term without the need for bat specific growth factors. Wnt, noggin and R-spondin are highly conserved in mammalian species, with a high degree of sequence identity between mice and JFBs. The growth requirements for our JFB organoids are consistent with growth conditions previously described for Chinese horseshoe bats ^25^ and Rousettus bats ^23^. Similar culture conditions also have been successfully used to culture intestinal organoids from cat, dog, cow, horse, pig and sheep ^22^ We demonstrate that JFB organoids from stomach, proximal and distal small intestine recapitulate the histology and morphology of the tissue of origin, with polarized columnar epithelial cells, mucus secretion, development of an intact epithelial barrier and expression of tissue-specific genes. Thus, we have developed and validated a new research tool that will allow experimental analysis of the physiology and function of the gastrointestinal epithelium of Jamaican fruit bats in future studies.

To summarize, we established and characterized JFB gastrointestinal organoids that recapitulated the organ-specific multicellular composition of JFB gastrointestinal tissue. We demonstrated SARS-CoV-2 sgRNA replication at a low efficiency in JFB distal intestinal organoids via qPCR but were unable to detect release of infectious virus. SARS-CoV-2 infection induced a robust upregulation of interferons and proinflammatory genes in the organoid cells. Moreover, SARS-CoV-2 infection of JFB organoids led to increased growth and activation of cellular regeneration and healing pathways, which might contribute to the improved viral tolerance in this bat species.

## Materials And Methods

### Tissue samples.

Male and female Jamaican fruit bats (*Artibeus jamaicensis)* were maintained as a breeding colony in an AAALAC-acoredited facility at Colorado State University (CSU) under and approved Institutional Animal Care and Use Committee protocol (#1034). For organoid derivation, five adult bats (4 male, 1 female) were euthanized by 5% isoflurane in O_2_ followed by thoracotomy. The gastrointestinal tracts were harvested in RPMI-1640 medium and were shipped overnight on ice from CSU to Montana State University (MSU).

### Crypt and gland isolation methods.

Bat tissues were processed immediately upon arrival or were cryopreserved and then thawed rapidly if needed ^72^. To derive organoids, proximal intestinal and distal intestinal tissues were washed in cold PBS and cut into ~ 1 mm pieces. The minced tissue was incubated in 15 mM EDTA in PBS supplemented with, penicillin, streptomycin, and Fungizone (GE Healthcare Life Sciences) with gentle shaking for 10 min increments until crypts appeared in the supernatant. Large tissues pieces were removed by sedimentation. The supernatant containing the crypts was transferred into a new 50 mL tube and pelleted by centrifugation for 8 min at 150 *g*. Gastric tissues were digested using a digestion solution containing 5 U/mL collagenase type IV and 0.2 mg/mL DNAse (both Sigma-Aldrich), following our published protocols ^73, 74^. Recovered crypts/glands were resuspended in 10 μl of Matrigel and plated in 96-well plates. After the gel was polymerized, 200 μl of medium (**Supplemental Table 3**) was added, and the plates were incubated at 37°C with 5% CO_2_ for one week.

### Maintenance of JFB organoids.

For passaging, the Matrigel patties containing organoids were digested for 3 min in TrypLE (Gibco) at 37°C and pipetted up and down 50 times. The digested organoids were harvested by centrifugation for 5 min, 200 *g* at 4°C, then were resuspended Matrigel and plated in a 24-well plate. After the gel had polymerized, 500 μl of medium was added and the plate was incubated at 37°C with 5% CO_2_. The medium was changed every other day and the organoids were passaged every 5–7 days.

### Optimization of growth conditions.

In addition to the basic growth medium, termed L-WRN medium, described above, we also tested a commercially available growth medium, IntestiCult^™^ (StemCell), a complex medium termed “colonoid medium” described by Tsai *et al.* ^72^, and analyzed medium supplementation with a number of different growth factors commonly used in organoid culture protocols (**Supplemental Table 3**). We prepared a medium with all available growth factors (L-WRN Plus) and then eliminated one reagent at a time from L-WRN Plus to determine the influence of the reagent on organoid growth. For this assay, the organoids were digested with TrypLE for 3 min and plated in a 96-well plate with the different media. Cell viability and proliferation were measured using the CellTiter-Glo luminescence assay (Promega).

### Histological Analysis of JFB Organoid Cultures.

Organoids were recovered from the culture plates and treated with Histogel (ThermoFisher) prior to formalin fixation and paraffin embedding, following standard protocols. Slides were stained with hematoxylin/eosin and with Alcian Blue to visualize mucus production.

### SARS-CoV-2 Infection of JFB organoids.

Bat organoids were dissociated by incubation with 350 μL TrypLE to expose the apical and basolateral epithelial surface to the virus. Dissociated organoids were transferred to a BSL3 laboratory and then inoculated with SARS-CoV-2 (strain USA-WA1/2020, BEI Resources), at a multiplicity of infection (MOI) of 0.1, 1 and 10 for 2 h at 37°C with frequent gentle agitation. Notably, the SARS-CoV-2 strain used was shown to have a defective furin cleavage site ^75^, but readily infected inducible pluripotent stem cell-derived human intestinal organoids in control experiments. The infected bat organoids were incubated in 30 μL of DMEM at 37°C for 2 hours with occasional shaking. Organoids were collected into 500 μL DMEM and centrifuged at 200 *g*for 5 min to wash. Then cells were resuspended in 30 μL Matrigel and plated. After 10 min to allow gelation of the Matrigel, medium was added to the organoids. This medium was removed and fresh medium added to eliminate free viral particles. Then the plates were incubated at 37°C for the indicated intervals. Infectious particles in culture supernatants were detected for each time point by plaque assay on Vero E6 cells, as previously described ^76^.

### Treatment of JFB organoids with TLR agonists and inactivated virus.

To analyze transcriptional response of JFB organoids to stimulation with pathogen-associated molecular patterns, organoids were trypsinized and then re-embedded into Matrigel in the presence of the following TLR agonists (Human TLR1-9 agonist kit, InvivoGen): TLR1, Pam3CSK4, 1 μg/mL; TLR2, heat-killed *Listeria monocytogenes* (10^8^/mL); TLR3, low molecular weight poly I:C, 10 μg/mL; TLR7, imiquimod, 1 μg/mL; TLR9, ODN2006, 5 μM. Alternatively, organoids were treated with UV-inactivated SARS-CoV-2 ^76^ (10 μg/mL). After 48 h, Organoids were lysed in TRI Reagent (Sigma) and processed for RNA isolation and RT-PCR.

### Quantitative RT-PCR.

To analyze gene expression and cell-associated viral RNA, RNA was extracted from organoids using the Direct-zol RNA Miniprep-Plus (Zymo Research). The RNA was converted to cDNA using iScript Reverse Transcription Super mix for RT-qPCR (BioRad). Primers for gastric and intestinal epithelial cell-specific genes and cytokines were designed using NCBI primer blast using the JFB genome (*Artibeus jamaicensis*, textid: 9417) and are listed in **Supplemental Table 4**. GAPDH was amplified as housekeeping gene in each PCR reaction. For each gene, a standard curve was created, and gene copy numbers for each gene of interest were normalized to the copy numbers of the housekeeping gene, GAPDH. To quantify SARS-CoV-2 in the organoid supernatant, viral RNA was extracted from culture supernatants using the QIA^®^Amp Viral RNA Mini kit (Qiagen). Viral genomes were then quantified in a single step RT-PCR reaction using primers and a TaqMan probe to the SARS-CoV-2 envelope (E) gene, as previously described ^76^, and the Quanta Bio ToughMix Master Mix. In addition, a forward primer to the leader sequence was used together with the reverse primer and probe to detect E gene sgRNA as described by Wölfel *et al.* ^46^. An RNA standard curve generated from a T7 *in vitro* transcribed gBlock^™^ sequence (Integrated DNA Technologies) was used for normalization.

### Immunofluorescence Staining.

For visualization of epithelial cytokeratin, we used a mouse-anti cytokeratin antibody that detects cytokeratins in a wide range of species (Thermofisher, 50-191-151). For visualization of SARS-CoV-2 protein in the organoid cultures, a monoclonal antibody to SARS-CoV-2 (11G10-F8) was generated in house, using a standard hybridoma protocol ^77^. Briefly, mice were immunized with 10 μg UV-inactivated SARS-CoV-2 (USA-WA1/2020) ^76^ in Titermax adjuvant (Sigma) three times separated by at least two weeks. 11G10-F8 was then generated from a fusion of mouse splenocytes with SP2/0 cells. Mouse sera were screened for reactivity to the virus by ELISA. Clone 11G10-F8 recognizes the RBD region of the S1 subunit of the spike protein and was used at a concentration of 10 μg/mL. For immunofluorescence analysis, organoids were fixed with 4% PFA, permeabilized with 0.2% Triton X-100, and then treated with blocking buffer (DPBS with 10% FBS, 0.2% Triton X-100, 0.1 % BSA, and 0.05% Tween) overnight. After washing, samples were incubated with primary antibody for 2 hours at room temperature. Then the secondary antibodies (goat anti-mouse IgG (H + L) AlexaFluor 594, Invitrogen, A11005; or rat anti-mouse IgGI eFluor660, eBiosciences, 50-112-4348), were added at 1:100 and incubated for 2 hours at room temperature. The nuclei were stained with 5 μM DAPI (MP Biomedicals, 0215757405). Actin filaments were stained with ActinGreen 488 ReadyProbes reagent-(Invitrogen, R37110). Stained organoids were imaged on an inverted SP5 Confocal Scanning Laser Microscopy (Leica) with 405 nm, 488 nm, 561 nm and 633 nm laser excitation lines using a 20x objective (W 2010; Zeiss, Oberkochen, Germany). Z-stacks of 2–11 randomly selected organoids with intact morphology for each experiment and condition were recorded.

### Cell viability and organoid growth.

To measure caspase 3 activity in SARS-CoV-2-infected organoids, NucView488 (Biotium) was added to the medium at 3 μM once the organoids were re-plated following incubation with the virus. For measuring caspase-3 activity, the organoids were imaged using Life Technologies EVOS FL Auto system with a 10x objective. The images were analyzed using ImageJ version 1.48V and NucView positive pixels were counted automatically on the thresholded images. Brightfield images of the organoid cultures were used to measure organoid size for normalization of the NucView data and for assessment of organoid growth.

### Proteomics analyses

Triplicate samples of distal intestinal organoids were infected with SARS-CoV-2, MOI 10, for 48 h as described above and then were lysed in RIPA lysis buffer (25 mM Tris/Cl, 150 mM NaCl, 1% NP-40, 1% SDS, 1% protease inhibitor) by passing the samples through a 26.5G needle 5 times on ice. Samples were stored at −80° C until they were analyzed at the IDeA National Resource for Quantitative Proteomics. An Orbitrap Exploris 480 was used for data-independent acquisition (DIA) mass spectrometry with a 60 min gradient per sample and gas-phase fractionation to obtain comprehensive proteomic profiles of the organoids. Chromatogram libraries were constructed using Prosit ^78^, and proteins were identified and quantified using EncyclopeDIA, based on protein FASTA files retrieved from NCBI RefSeq for the Jamaican fruit bat (BioProject PRJNA673233) ^51, 52^. The mass spectrometry proteomics data have been deposited to the ProteomeXchange Consortium via the PRIDE ^79^ partner repository with the dataset identifier PXD036016. False discovery thresholds of 1% were applied. The ProteiNorm app was used to optimize data normalization ^80^, and Scaffold DIA (Proteome Software, Portland, OR) was used for visualization. The MS2 exclusive intensities were normalized using cyclic loess and linear models for microarray (limma) and lmfit with empirical Bayes smoothing was used for the analysis ^81^. Proteins with an FDR-adjusted *P*-value ≤ 0.05 and an absolute fold change ≥ 2 were considered significant. Ingenuity Pathway Analysis (Qiagen) and Enrichr ^54^ with combined score ranking (c = log(p) * z, where c = the combined score, *P*= Fisher exact test *P*-value, and z = z-score) were used to identify cellular signaling pathways. COVID-19 related gene sets identified by Enrichr were based on the “The COVID-19 Drug and Gene Set Library, 2021 version” website ^82^. To analyze the impact of SARS-CoV-2 infection on ISGs, proteins identified in the JFB organoids were compared to a comprehensive list of ISGs ^53^ using a Python script.

## Data Availability

The mass spectrometry proteomics data have been deposited to the ProteomeXchange Consortium via the PRIDE ^79^ partner repository with the dataset identifier PXD036016.
